# Factors associated with type 2 diabetes in patients with vascular dementia: a population-based cross-sectional study

**DOI:** 10.1186/s12902-018-0273-z

**Published:** 2018-07-04

**Authors:** Chun-Lin Liu, Ming-Yen Lin, Shang-Jyh Hwang, Ching-Kuan Liu, Huei-Lan Lee, Ming-Tsang Wu

**Affiliations:** 1 Graduate Institute of Medicine, College of Medicine, Kaohsiung Medical University, No. 100, Shih-Chuan 1st Road, Kaohsiung, 80708 Taiwan; 2 Calo Psychiatric Center, No.12-200, Jinhua Rd., Xinpi Township, Pingtung County 925 Taiwan; 3 Department of Renal Care, College of Medicine, Kaohsiung Medical University, No. 100, Shih-Chuan 1st Road, Kaohsiung, 80708 Taiwan; 4 Division of Nephrology, Department of Internal Medicine, Kaohsiung Medical University Hospital, Kaohsiung Medical University, No. 100, Shih-Chuan 1st Road, Kaohsiung, 80708 Taiwan; 5 Research Center for Environmental Medicine, Kaohsiung Medical University, No. 100, Shih-Chuan 1st Road, Kaohsiung, 80708 Taiwan; 6 Institute of Population Health Sciences, National Health Research Institutes, No. 35 Keyan Road, Zhunan, Miaoli, 35053 Taiwan; 7 Department of Neurology, Kaohsiung Medical University Hospital, Kaohsiung Medical University, No. 100, Shih-Chuan 1st Road, Kaohsiung, 80708 Taiwan; 8 Department of Public Health, Kaohsiung Medical University, No. 100, Shih-Chuan 1st Road, Kaohsiung, 80708 Taiwan; 9 Graduate Institute of Clinical Medicine, Kaohsiung Medical University, No. 100, Shih-Chuan 1st Road, Kaohsiung, 80708 Taiwan

**Keywords:** Type 2 diabetes, Comorbidity, Dementia, Socioeconomic status

## Abstract

**Background:**

Incidence of dementia is growing rapidly and affects many people worldwide. Type 2 diabetes mellitus (DM) might link cognitive decline and dementia, but the reasons for this association remain unclear. Our study explored the factors associated with type 2 DM in patients with dementia.

**Methods:**

Patients (*n* = 40,404) with vascular dementia were identified in Taiwan’s 1997 to 2008 National Health Insurance Research Database and divided into a DM group and non-DM group. Eleven comorbidities were identified and categorized into four groups: cardiovascular and cerebrovascular diseases, digestive system diseases, renal and metabolic system diseases, and cancer. The associations of these factors with type 2 DM were explored through multivaraible logistic regression.

**Results:**

Of the patients with dementia, 22.5% had DM. Associated with a higher likelihood of DM in this population were female sex (adjusted odds ratio [OR]: 1.44, 95% confidence interval [CI]: 1.36–1.52), young age (range of adjusted OR: 0.55–1.13), low income (range of adjusted OR: 1.09–1.18), and renal and metabolic system diseases (OR: 2.81, 95% CI: 2.64–2.98).

**Conclusions:**

The findings of this study suggest that clinicians should encourage patients with dementia to receive regular glucose impairment screening if they are female, have low socioeconomic status, or have renal or metabolic diseases.

**Electronic supplementary material:**

The online version of this article (10.1186/s12902-018-0273-z) contains supplementary material, which is available to authorized users.

## Background

Dementia, including Alzheimer’s disease and vascular dementia (VaD), is a progressive neurodegenerative disease affecting more than 35 million people worldwide [1]. It can shorten human life, reduce patient and caregiver quality of life, and cause substantial economic burden [2–4]. As the number of patients with dementia increases, understanding the natural course of dementia is crucial for identifying vulnerable populations and appropriate interventions.

Type 2 diabetes mellitus (DM), which is found in 13–20% of patients with dementia [5], is a major disease linked with cognitive decline and dementia [6]. However, the reasons for this close association remain unclear. Through poorly controlled blood sugar, DM may damage blood vessels and produce long-term complications such as cerebrovascular disease, cardiovascular disease, or hypertension, all of which further accelerate progression toward cognitive impairment [7]. Following the interrelationships between DM, rare complications, and dementia may be possible by tracing large DM populations over a long period. Until then, the prevalences of DM related comorbidities in the dementia population should be determined to help identify patients at high risk, and cognitive impairment screening is required to help physicians prevent or treat DM-related complications that can lead to dementia. To begin this line of research, we conducted a population-based cross-sectional study to estimate the prevalence of type 2 DM in patients with dementia and explored factors associated with the development of DM in the study population.

## Methods

### Data source

The Department of Health in Taiwan implemented the National Health Insurance (NHI) program in 1995. By the end of 1996, approximately 96% of all residents in Taiwan had enrolled [8]. The coverage rate increased steadily from 96.1 to 98.6% from 2000 to 2007. All hospitals and clinics contracted with the NHI program are required to submit patient claims to receive reimbursements from the program. To verify the accuracy of claims data, the Bureau of NHI Management performs quarterly expert reviews on a random sample of inpatient claims at each hospital and clinic. Severe penalties are issued for false reports. We linked data from Taiwan’s Registry for Catastrophic Illness Database and related outpatient and inpatient claims datasets from 1997 to 2008.

### Study design and population

For this population-based cross-sectional study, we included all patients in Taiwan with dementia who were newly registered to receive the catastrophic illness certification for senile dementia according to the International Classification of Diseases, Ninth Revision, Clinical Modification (ICD-9-CM; code 290.X) from 1997 to 2008. These designations are based on rigid diagnostic criteria evaluated by a neurologist or psychiatrist. The registry date found on the catastrophic illness certification was considered the index date for this study. All patients were defined as having comorbid diabetes if diagnostic ICD-9-CM code 250.XX appeared on at least two ambulatory care claims records or at least one inpatient care claims record within 1 year leading up to dementia diagnosis.

### Assessment of associated factors

Several factors were considered to assess their potential associations with type 2 DM, namely demographic factors (age, sex, area of residence, urbanization level, and insurance amount), comorbidities (myocardial infarction, congestive heart failure, peripheral vascular disease, cerebrovascular disease, chronic pulmonary disease, peptic ulcer disease, mild liver disease, renal disease, cancer, hypertension, and hyperlipidemia), and disease severity. Area of residence was classified into four regions of Taiwan (north central, south, and east); urbanization level was divided into urban and rural; and insurance amount was divided into dependent, < NT$20,000 per month, and ≥ NT$20,000 per month. Patients were defined as “low income” when their insurance amount was dependent, “medium income” when their insurance amount was < NT$20,000 per month, and “high income” when their insurance amount was ≥ NT$20,000 per month. Comorbidities were defined based on ICD-9-CM codes (Additional file 1: Table S1) listed on two or more ambulatory care claims records or one or more inpatient care claims records during the year prior to dementia diagnosis. To further evaluate the associations of various systematic diseases with diabetes, we grouped these comorbidities into four main categories based on similar manageable risk factors. These categories were (1) cardiovascular and cerebrovascular diseases (myocardial infarction, congestive heart failure, peripheral vascular disease, cerebrovascular disease, chronic pulmonary disease, and hypertension), (2) digestive system diseases (peptic ulcer disease and mild liver disease), (3) renal and metabolic system diseases (renal disease and hyperlipidemia), and (4) cancer. Disease severity was defined based on Charlson comorbidity index scores, which are listed in a previous report [9], calculated based on diseases.

### Statistical analysis

Participant characteristics were analyzed as follows. Distributions of continuous variables were expressed as mean ± standard deviation (SD) or median (interquartile range), and those of categorical variables were expressed as numbers and percentages. The differences in the distributions of count variables between DM and non-DM were analyzed through Mann–Whitney *U* testing, and those of categorical variables were analyzed through the chi-squared (*χ*^2^) testing. Multivariable logistic regressions adjusted for all demographic factors, and comorbidities or various systematic diseases were tested to identify independent factors associated with DM. In addition, we investigated associations among various systematic disease groups, the number of each systematic disease, and DM by using multivariable logistic regression adjusted for patient characteristics. Data were represented as odds ratios (ORs) and 95% confidence intervals (CIs). To further explore the effects of income and comorbidities on the prevalence of DM, the proportion of DM and high income by number of comorbidities, and the proportions of various systematic diseases by DM and income were also investigated. To validate our main findings, we conducted sensitivity analysis after redefining the DM group as ICD-9-CM code 250.XX appearing on at least three ambulatory care claims records or at least one inpatient care claims record within 1 year leading up to dementia diagnosis. All statistical operations were performed in SAS (version 9.4, SAS Institute, Cary, NC, USA). A *p* value of < 0.05 was considered significant.

## Results

### Patient characteristics

In the study cohort, 22.5% patients with dementia also had diabetes. Table 1 provides a summary of patient characteristics. Compared with patients without diabetes, those with diabetes were significantly more likely to be female, young, and living in south and rural areas (Table 1). They were also more likely to have low incomes, more comorbidities (myocardial infarction, congestive heart failure, peripheral vascular disease, cerebrovascular disease, chronic pulmonary disease, peptic ulcer disease, mild liver disease, renal disease, cancer), and a greater number of severe diseases. The top three largest differences in the distributions of comorbidities between the DM and non-DM groups were cerebrovascular disease (percentage difference: 18.9), peptic ulcer disease (percentage difference: 7.1), and renal disease (percentage difference: 5.7).

**Table 1:** Patient Characteristics

	DM		Non-DM		*P*-value
	N	%	N	%	
Gender					
Male	3261	35.9	13,830	44.2	< 0.001
Female	5816	64.1	17,484	55.8	
Age (mean ± SD), yr	75.7 ± 8.1		77.1 ± 9.4		< 0.001
< 65	843	9.3	2878	9.2	< 0.001
65–74	3088	34.0	8165	26.1	
75–84	4149	45.7	14,584	46.5	
≥ 85	997	11.0	5687	18.2	
Area of residence					
North	3237	35.7	12,357	39.4	< 0.001
Central	2192	24.1	7310	23.3	
South	3269	36.0	10,287	32.9	
East	379	4.2	1360	4.4	
Urbanization level					
Urban	2837	31.2	10,419	33.3	< 0.001
Rural	6240	68.8	2,0895	66.7	
Insurance amount, NT$/month					
Dependent	4522	49.8	13,554	43.3	< 0.001
< 20,000	3013	33.2	12,043	38.4	
≥ 20,000	1542	17.0	5717	18.3	
Charlson comorbidity index					
Mean ± SD	3.9 ± 1.9		1.7 ± 1.5		< 0.01
Median (Interquartile range)	4.0 (2.0–5.0)		1.0 (1.0–2.0)		< 0.01
Selective comorbidities					
Myocardial infarction	176	1.9	359	1.2	< 0.01
Congestive heart Failure	1071	11.8	2219	7.1	< 0.01
Peripheral vascular disease	317	3.5	671	2.1	< 0.01
Cerebrovascular disease	4499	49.6	9608	30.7	< 0.01
Chronic pulmonary disease	1969	21.7	5593	17.9	< 0.01
Peptic ulcer disease	1800	19.8	3981	12.7	< 0.01
Mild liver disease	790	8.7	1601	5.1	< 0.01
Renal disease	855	9.42	1166	3.7	< 0.01
Cancer	499	5.5	1250	4.1	< 0.01
Hypertension	6537	72.0	12,669	40.5	< 0.01
Hyperlipidemia	1854	20.4	1791	5.72	< 0.01

*DM*, diabetes mellitus, *DM; NT$,* New Taiwan dollar

Differences in characteristics between the DM and non-DM groups were tested through the Mann–Whitney *U* test or *χ*^2^ test; *p* < 0.05 was considered significant

### Factors related to DM prevalence

**Fig. 1:**
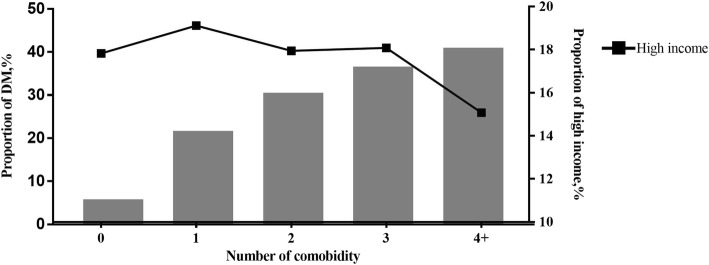
Proportion of patients with diabetes and high income by number of comorbidities. High income is defined by monthly insurance amount of ≥NT$20,000

**Fig. 2:**
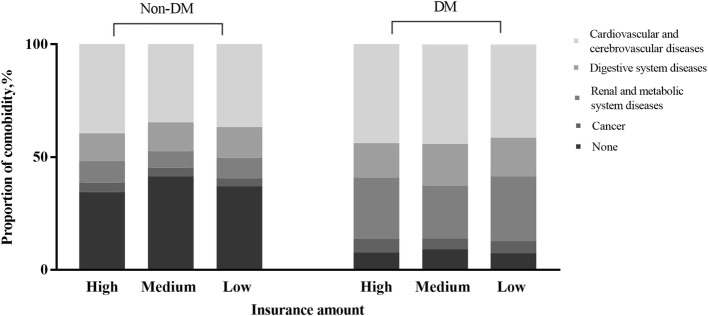
Proportion of comorbidity-related diseases by diabetes and insurance amount. High, middle, and low income are defined by insurance amount(high: ≥NT$20,000 monthly, medium: < NT$20,000 monthly, and low: dependent)

Table 2 shows the associations of baseline characteristics and comorbidities with DM. Age, sex, area of residence, urbanization level, insurance amount, congestive heart failure, cerebrovascular disease, peptic ulcer disease, mild liver disease, renal disease, hypertension, and hyperlipidemia were significantly and independently associated with DM in patients with dementia (Table 2). Notably, patients with DM were more likely to be female (adjusted OR: 1.44, 95% CI: 1.36–1.52) and young (range of adjusted OR: 0.55–1.13). Those with DM were significantly more likely to have more comorbidities, have lower incomes, and live in rural areas. We analyzed the associations of DM with income, number of comorbidities, and systematic diseases (Figs. 1 and 2). As can be seen in Fig. 1, which shows the results of our analysis regarding the prevalence of DM, number of comorbidities, income, the prevalence of DM was higher in patients with four or more comorbidities and lower in those with high incomes (Fig. 1). Figure 2 depicts the results of our analysis regarding the proportion of comorbidity-related diseases based on diabetes status and insurance amount. Patients with DM had substantially more comorbidities than did those without DM. Those with DM and those in the lower income group had the highest proportion of renal and metabolic system diseases (Fig. 2). The sensitivity analysis strongly supported these model findings (Additional file 1: Table S2).

**Table 2:** Associations of baseline characteristics and comorbidities with type 2 diabetes mellitus

Parameters	Multivariable-adjusted model	
	Odds ratio	95%CI
Gender		
Male	1.00 [Reference]	
Female	1.44	1.36–1.52
Age, yr		
< 65	1.00 [Reference]	
65–74	1.13	1.02–1.24
75–84	0.86	0.78–0.95
≥ 85	0.55	0.49–0.62
Area of residence		
North	1.00 [Reference]	
Central	1.14	1.06–1.22
South	1.12	1.06–1.19
East	1.17	1.05–1.19
Urbanization level		
Urban	1.00 [Reference]	
Rural	1.12	1.06–1.19
Insurance amount, NT$/month		
≥ 20,000	1.00 [Reference]	
< 20,000	1.09	1.09–1.27
Dependent	1.18	1.01–1.17
Selected comorbidities		
Myocardial infarction	1.01	0.83–1.23
Congestive heart failure	1.12	1.06–1.26
Peripheral vascular disease	1.12	0.99–1.34
Cerebrovascular disease	1.56	1.48–1.64
Chronic pulmonary disease	1.03	0.97–1.09
Peptic ulcer disease	1.25	1.17–1.34
Mild liver disease	1.42	1.28–1.56
Renal disease	2.00	1.81–2.21
Cancer	1.27	1.13–1.42
Hypertension	2.79	2.65–2.95
Hyperlipidemia	2.93	2.72–3.15

*CI*, confidence interval; *NT$*, New Taiwan dollar

Multivariable logistic regressions adjusted for sex, age, income, area of residence, urbanization level, and comorbidities were run to identify independent factors associated with type 2 diabetes mellitus; *p* < 0.05 was considered significant

### Systematic diseases and DM prevalence

Table 3 shows the results of our study regarding association between systematic comorbidities and DM in patients with dementia. Prevalence of DM was significantly associated with the following systematic comorbidities: cardiovascular diseases (adjusted OR: 3.93, 95% CI: 3.68–4.19); digestive system diseases (adjusted OR: 1.34, 95% CI: 1.26–1.42); renal and metabolic system diseases (adjusted OR: 2.81, 95% CI: 2.64–2.98); and cancer (adjusted OR: 1.23, 95% CI: 1.09–1.38) (Table 3). The likelihood of DM was clearly higher in patients with one or two renal or metabolic system diseases than in those without (adjusted OR: 3.05, 95% CI: 2.86–3.25 and adjusted OR: 5.32, 95% CI: 4.18–56.78, respectively). Similar results from the sensitivity analysis are shown in Additional file 1: Table S3.

**Table 3:** Associations of systemic comorbidities with type 2 diabetes mellitus

Parameters	Univariable model		Multivariable-adjusted model^d^	
	OR	95%CI	OR	95%CI
Cardiovascular and cerebrovascular diseases^a^				
No	1.00 [Reference]		1.00 [Reference]	
Yes	4.67	4.38–4.98	3.93	3.68–4.19
Number				
0	1.00 [Reference]		1.00 [Reference]	
1–3	2.57	2.45–2.69	2.28	2.17–2.41
4–6	2.81	2.39–3.29	2.34	1.97–2.76
Digestive system diseases^b^				
No	1.00 [Reference]		1.00 [Reference]	
Yes	1.79	1.69–.189	1.34	1.26–1.42
Number				
0	1.00 [Reference]		1.00 [Reference]	
1	1.78	1.67–1.88	1.43	1.35–1.53
2	1.93	1.66–2.25	1.55	1.32–1.82
Renal and metabolic system diseases^c^				
No	1.00 [Reference]		1.00 [Reference]	
Yes	3.85	3.63–4.09	2.81	2.64–2.98
Number				
0	1.00 [Reference]		1.00 [Reference]	
1	3.72	3.49–3.95	3.05	2.86–3.25
2	7.07	5.60–8.93	5.32	4.18–6.78
Cancer				
No	1.00 [Reference]	1.00 [Reference]		
Yes	1.39	1.26–1.56	1.23	1.09–1.38

*OR*, odds ratio; *CI*, confidence interval

^a^Cardiovascular and cerebrovascular diseases comprised myocardial infarction, congestive heart failure, peripheral vascular disease, cerebrovascular disease, chronic pulmonary disease, and hypertension

^b^Digestive system diseases comprised peptic ulcer disease and mild liver disease

^c^Renal and metabolic system diseases comprised renal disease and hyperlipidemia

^d^Multivariable logistic regressions adjusted for sex, age, income, area of residence, and urbanization, and various systematic comorbidities were run to identify independent factors associated with type 2 diabetes mellitus; *p* < 0.05 was considered significant

## Discussion

Although epidemiology studies have reported that type 2 DM is associated with an increased risk of Alzheimer’s disease and VaD [10], few studies have focused on the prevalence of type 2 DM in patients with dementia. The present population-based cross-sectional study found several factors independently associated with high prevalence of diabetes in patients with dementia. We found that likelihood of DM was higher in female, young, and low-income patients as well as those with one or more comorbidities and those with both hyperlipidemia and renal disease.

More than one-fifth of the patients with dementia had type 2 DM, suggesting that improved glucose management is required during the predementia phase. Previous studies have suggested that in the population with DM, long-term poor DM management and repeated serious glycemic episodes result in cognitive impairment in older adults [11, 12]. Consistent with one previous report [13], in the present study, female sex was associated with an increased likelihood of having DM with dementia. We also found that the patients with dementia who had DM were nearly 2 years younger on average than those without DM. This finding is similar to a prior observation in an Australian population [14] and might suggest a need for more targeted blood glucose management for women, considering other targeting criteria.

Previous studies have found high prevalences of comorbid medical conditions and related complications in patients with dementia. In a recent review of 54 primary studies, Bunn et al. concluded that DM and stroke were the most prevalent comorbid conditions in patients with dementia (13–20% and 16–29%, respectively) [15]. Another study identified 12 chronic commodities associated with dementia, most of which have similar pathophysiologies [5]. These chronic diseases may share similar risk factors or etiologies. Although anthropometric, dietary, and lifestyle factors were associated with type 2 DM in the general population [16], measurements of these risk factors were not easily obtained from patients with cognitive disorders. Identification of disease factors associated with type 2 DM may be more intuitive and more efficient than that of traditional type 2 DM risk factors for physicians providing optimal DM care for patients with dementia. The current study found that comorbidities with similar manageable risk factors, including cerebrovascular disease, renal disease, hypertension, and hyperlipidemia, were more likely to be grouped with DM in our study population, thereby emphasizing the need for comanagement of related metabolic system diseases to prevent DM-related complications in dementia.

This study found that lower income was associated with a higher prevalence of DM; the mechanisms underlying this association may be complex. A plausible explanation is that low-income patients tend to exert poor control over their glycemic levels, putting themselves at excessive risk of cardiovascular disease and accelerating progress toward cognitive dysfunction [17]. Alongside income differences may come differences in lifestyle factors, including education, occupation, and leisure activities, which have been increasingly recognized as factors that may affect the development of dementia [18–20]. Although having a privileged socioeconomic background might be associated with protection of brain function after damage, which reduces the risk of senile dementia [21], its potential moderation of the relationship between DM and dementia requires further study.

Several limitations of this study should be declared. It is unlikely, but possible, that the observed associations between diseases and DM resulted from more effective treatments and higher disease awareness among physicians and patients in the DM group compared with those in the non-DM group. Because of the long development from cognition impairment to dementia, we were unable to obtain the time of dementia onset. We were also unable to accurately differentiate types of dementia based on only diagnosis codes. Given that the study population was old and had high prevalence of chronic conditions, most of our patients likely had VaD. Finally, we were unable to control various potential confounders associated with DM and dementia, including educational status, life habits, and clinical laboratory data, that may have contributed to our findings. These factors may be helpful for clarifying the interrelationships between type 2 DM, comorbidities, and dementia in further research.

## Conclusions

This study found significant associations of gender, socioeconomic status, and comorbidities with DM in patients with dementia. Based on our findings, patients with dementia who are female, have low income, and have comorbid renal or metabolic disease should undergo routine glucose impairment examinations to facilitate the management of blood glucose and prevention of adverse glycemic events.

## Additional file

Additional file 1: **Table S1.** Diseases and corresponding ICD-9-CM codes. **Table S2.** Associations of baseline characteristics and comorbidities with diabetes mellitus. **Table S3.** Associations of systemic comorbidities with diabetes mellitus. (DOCX 32 kb)

## Abbreviations

CI: Confidence Interval
DM: Diabetes Mellitus
ICD-9-CM: International Classification of Disease, Ninth Revision, Clinical Modification
NHI: National Health Insurance
NT$: New Taiwan Dollar
OR: Odds Ratio
VaD: Vascular Dementia
