# Supplementary material for: Factors associated with type 2 diabetes in patients with vascular dementia: a population-based cross-sectional study
Source: BMC Endocr Disord. 2018 Jul 4;18:45. doi: 10.1186/s12902-018-0273-z (PMC6033220; doi:10.1186/s12902-018-0273-z)
Supplement: Supplementary file 1 — Table S1. Diseases and corresponding ICD-9-CM codes. Table S2. Associations of baseline characteristics and comorbidities with diabetes mellitus. Table S3. Associations of systemic comorbidities with diabetes mellitus. (DOCX 32 kb) [file 12902_2018_273_MOESM1_ESM.docx]

Table S1. Diseases and corresponding ICD-9-CM codes.

| Co-morbidity | Corresponding ICD-9-CM Codes |
| --- | --- |
| Myocardial Infarction | 410, 412 |
| Congestive Heart Failure | 39891, 40201, 40291, 40401, 40403, 40411, 40413, 40491, 40493, 4254, 4255, 4257, 4258, 4259, 428 |
| Peripheral Vascular Disease | 0930, 4373, 440, 441, 4431, 4432, 4438, 4439, 4471, 5571, 5579 |
| Cerebrovascular Disease | 36234, 430, 431, 432, 433, 434, 435, 436, 437, 438 |
| Chronic Pulmonary Disease | 4168, 4169, 490-496, 500-505, 5064, 5081, 5088 |
| Peptic Ulcer Disease | 531, 532, 533, 534 |
| Mild liver disease | 07022, 07023, 07032, 07033, 07044, 07054, 0706, 0709, 570, 571, 5733, 5734, 5738, 5739, V427 |
| Renal Disease | 40301, 40611, 40391, 40402, 10403, 40412, 40413, 40492, 40493, 582, 5830, 5831, 5832, 5834, 5836, 5837, 585, 586, 5880, V420, V451, V56 |
| Cancer | 140-195, 200-208, 2386 |
| Hypertension | 401, 402, 403, 404, 405 |
| Hyperlipidemia | 272 |

Abbreviation: ICD-9-CM, International Classification of Diseases, Ninth Revision, Clinical Modification.

| Table S2. Associations of baseline characteristics and comorbidities with diabetes mellitus. | | |
| --- | --- | --- |
| Parameters | Multivariable-adjusted model | |
|  | Odds ratio | 95%CI |
| Gender |  |  |
| Male | 1.00 [Reference] | |
| Female | 1.44 | 1.36-1.52 |
| Age |  |  |
| <65 | 1.00 [Reference] | |
| 65-7~~4~~ | 1.13 | 1.03-1.24 |
| 75-84 | 0.85 | 0.78-0.94 |
| ≥85 | 0.55 | 0.49-0.62 |
| Area of residence |  |  |
| North | 1.00 [Reference] | |
| Central | 1.15 | 1.07-1.24 |
| South | 1.13 | 1.06-1.20 |
| East | 1.19 | 1.04-1.36 |
| Urbanization level |  |  |
| Urban | 1.00 [Reference] | |
| Rural | 1.13 | 1.03-1.20 |
| Insurance amount, NT$/month |  |  |
| ≥20,000 | 1.00 [Reference] | |
| <20,000 | 1.07 | 0.99-1.16 |
| Dependent | 1.17 | 1.08-1.26 |
| Selected comorbidities |  |  |
| Myocardial infarction | 1.05 | 0.86-1.28 |
| Congestive heart failure | 1.17 | 1.07-1.27 |
| Peripheral vascular disease | 1.17 | 1.01-1.35 |
| Cerebrovascular disease | 1.58 | 1.49-1.67 |
| Chronic pulmonary disease | 1.01 | 0.95-1.08 |
| Peptic ulcer disease | 1.26 | 1.18-1.35 |
| Mild liver disease | 1.38 | 1.25-1.52 |
| Renal disease | 2.04 | 1.85-2.25 |
| Cancer | 1.28 | 1.14-1.44 |
| Hypertension | 2.81 | 2.66-2.97 |
| Hyperlipidemia | 2.87 | 2.66-3.09 |

Diabetes was defined as ICD-9-CM code 250.XX appearing on at least three ambulatory care claims records or at least one inpatient care claims record with 1 year leading up to dementia diagnosis.

Abbreviations: CI, confidence interval; NT$, New Taiwan dollar.

Logistic regression analysis was conducted for odds ratio estimation after adjustment for sex, age, insurance amount, area of residence, urbanization level, and comorbidities; *p* < 0.05 was considered significant.

| Table S3. Associations of systemic comorbidities with diabetes mellitus. | | | | | | | | | | |  |
| --- | --- | --- | --- | --- | --- | --- | --- | --- | --- | --- | --- |
| Parameters | Univariable model | |  | | Multivariable-adjusted model^*^ | | | | | |  |
|  | OR | 95%CI | |  | | OR | | | | 95%CI | |
| **Cardiovascular and cerebrovascular diseases^1^** | |  | |  | | |  | | | | |
| No | 1.00 [Reference] | | |  | | | 1.00 [Reference] | | | | |
| Yes | 4.67 | 4.38-4.98 | |  | | | 3.93 | | 3.68-4.19 | | |
| **Number** |  | | |  | | |  | | | | |
| 0 | 1.00 [Reference] | | |  | | | 1.00 [Reference] | | | | |
| 1-3 | 2.57 | 2.44-2.69 | |  | | 2.29 | | | | 2.17-2.41 | |
| 4-5 | 2.81 | 2.39-3.29 | |  | | 2.34 | | | | 1.97-2.76 | |
| **Digestive system diseases^2^** |  | | |  | | |  | | | | |
| No | 1.00 [Reference] | | |  | | | 1.00 [Reference] | | | | |
| Yes | 1.79 | 1.69-1.89 | |  | | | 1.34 | 1.26-1.42 | | | |
| **Number** |  |  | |  | | |  |  | | | |
| 0 | 1.00 [Reference] | | |  | | | 1.00 [Reference] | | | | |
| 1 | 1.78 | 1.67-1.86 | |  | | 1.43 | | | | 1.35-1.53 | |
| 2 | 1.93 | 1.66-2.25 | |  | | 1.55 | | | | 1.32-1.82 | |
| **Renal and metabolic system diseases^3^** |  | | |  | | |  | | | | |
| No | 1.00 [Reference] | | |  | | | 1.00 [Reference] | | | | |
| Yes | 3.85 | 3.63-4.09 | |  | | | 2.81 | 2.64-2.99 | | | |
| **Number** |  |  | |  | | |  |  | | | |
| 0 | 1.00 [Reference] | | |  | | | 1.00 [Reference] | | | | |
| 1 | 3.72 | 3.49-3.95 | |  | | 3.05 | | | | 2.86-3.25 | |
| 2 | 7.07 | 5.50-8.93 | |  | | 5.32 | | | | 4.19-6.78 | |
| **Cancer** |  |  | |  | |  | | | |  | |
| No | 1.00 [Reference] | |  | | 1.00 [Reference] | | | | | | |
| Yes | 1.39 | 1.26-1.56 | |  | | 1.23 | | | | 1.09-1.38 | |
| Diabetes was defined as ICD-9-CM code 250.XX appearing on at least three ambulatory care claims records or at least one inpatient care claims record within 1 year leading up to dementia diagnosis.  Abbreviations: OR, odds ratio; CI, confidence interval. | | | | | | | | | | |  |
| ^1^Cardiovascular and cerebrovascular diseases comprised myocardial infarction, congestive heart failure, peripheral vascular disease, cerebrovascular disease, chronic pulmonary disease, and hypertension.  ^2^Digestive system diseases comprised peptic ulcer disease and mild liver disease.  ^3^Renal and metabolic system diseases comprised renal disease and hyperlipidemia.  *Logistic regression analysis was used for odds ratio estimation after adjustment for sex, age, income, area of residence, urbanization level, and various systematic comorbidities; *p* < 0.05 was considered significant. | | | | | | | | | | |  |
